# Influence of Cross-Linking and Crystalline Morphology on the Shape-Memory Properties of PET/PEN/PCL Copolyesters Using Trimesic Acid and Glycerol

**DOI:** 10.3390/polym15092082

**Published:** 2023-04-27

**Authors:** Fu-Ting Yang, Yu-Ming Chen, Syang-Peng Rwei

**Affiliations:** 1 Institute of Organic and Polymeric Materials, National Taipei University of Technology, No. 1, Sec. 3, Chung-Hsiao East Road, Taipei 10608, Taiwan; 2 Taiwan Textile Research Institute, No. 6, Chengtian Road, Tucheng Dist., New Taipei City 23674, Taiwan; t7350304@gmail.com; 3 Research and Development Center of Smart Textile Technology, National Taipei University of Technology, No. 1, Sec. 3, Chung-Hsiao East Road, Taipei 10608, Taiwan

**Keywords:** poly(ethylene terephthalate), polycaprolactone, poly(ethylene naphthalate), cross-link, shape memory

## Abstract

PCL-based biodegradable shape-memory polymers (SMPs) are limited in strength, which restricts their practical applications. In this study, a series of novel SMPs, composed of poly(ethylene terephthalate) (PET), poly(ethylene naphthalate) (PEN), and poly(ε-caprolactone) (PCL), were synthesized and cross-linked using planar (benzene-1,3,5-tricarboxylic acid, BTC) or non-planar (glycerol, GC) cross-linkers via the one-pot method. The influence of different kinds of cross-linkers and hard segments of copolyesters on the thermal properties, crystallization behavior, mechanical properties, shape-memory performance, and degradability was investigated by FT-IR, ^1^H-NMR, DSC, DMA, TGA, XRD, tensile test, intrinsic viscosity measurement, and in vitro enzymatic degradation test. The results indicate that the tensile strength of the copolyester can be significantly improved from 27.8 to 53.2 MPa by partially replacing PET with PEN while maintaining its shape-memory characteristics. Moreover, a small amount of cross-linking modification leads to higher temperature sensitivity, improved shape recovery rate at third round (R_r_(3) = 99.1%), and biodegradability in the cross-linked PET/PEN/PCL shape-memory polymers. By changing the crystallization morphology and cross-linking forms of the material, we have developed a shape-memory polymer with both high strength and a high shape recovery rate, which provides a new strategy for the development of shape-memory materials.

## 1. Introduction

Benefiting from lightweight and tunable recoverability, shape-memory polymers (SMPs) are an emerging class of polymers that are attracting much attention from various fields [1,2,3,4]. They can be deformed from their original shape to one or more temporary shapes by mechanical deformation and subsequent fixation of that deformation at ambient temperatures without external force. Once specific stimuli are applied, SMPs are capable of recovering to their original shape, namely their permanent shape, also without external force. Among these stimuli, such as heat, light, solvent, electricity, magnetism, pH, and moisture, there is most focus on the heat-induced SMP [5,6,7,8]. Because the shape-memory effect is an entropy-driven recovery of mechanical deformation and only relies on the molecular architecture, which does not require a specific chemical structure in the repeating units [4,9], many researchers are committed to developing various formulae, by studying their shape-memory properties, and trying to summarize the relationship between molecular architecture and shape-memory properties [9,10,11,12].

Polyethylene terephthalate (PET) is one of the most critical commercial thermoplastic materials. As a semi-crystalline polymer, it possesses excellent thermal stability, chemical resistance, melt mobility, and spinnability and is widely used in packaging, electrical appliances, automobiles, construction, and other fields. Among PET applications, synthetic fibers account for the largest consumption [13,14,15,16]. However, as environmental protection becomes more and more important, non-biodegradability threatens its current application and future development. The most frequently and widely used methods involve incorporating biodegradable units, such as aliphatic esters, into the PET backbone through copolymerization or blending methods [17,18].

Poly(ε-caprolactone), PCL, is a synthetic, semi-crystalline, linear, and hydrophobic polyester that is well known for its non-toxicity, biodegradability, and biocompatibility. It has been approved by the U.S. Food and Drug Administration for use in the human body [19,20,21]. Because of its high crystallinity (Xc > 50%) and slow in vivo hydrolysis rate, it exhibits good mechanical properties in bulk, as well as in the form of copolymers and polymer nanocomposites [22,23,24].

Over the past two decades, PCL has been increasingly applied in medical devices, drug delivery systems, and tissue engineering, owing to its slow hydrolytic degradation rate in the human body and its suitable shape-memory effect, as well as that of its copolymers or blends [25,26,27,28]. A large variety of copolymers of PCL applied to shape-memory materials have been reported. For example, Zhu et al. [29] synthesized PCL-based PU by replacing 6–8 wt% 1,4-BDO with N-methyldiethanolamine (NMDA) or N,N-bis(2-hydroxyethyl)isonicotinamide (BIN) as an extender, and the corresponding cationomers were synthesized by neutralization of NMDA or BIN with a stoichiometric amount of acetic acid. The introduction of NMDA and BIN as cationomers resulted in improved crystallizability and shape-memory properties. Rabani et al. [30] synthesized SMP containing aramid hard segments by reacting a diamine with PCL-diol. Even at low hard segment content (less than 15 wt%), these aramid SMPUs were capable of providing effective physical cross-linking. Zhu et al. [31] investigated the influence of the molecular weights of PCL-diol on the crystallization behavior of segmented PU. The results indicate that the crystallization rate improved as the molecular weight of PCL increased. Zhu et al. [32] investigated the shape-memory effect of γ-irradiation on cross-linked PCL. The cross-linked PCL exhibited higher heat resistance and tensile modulus. Blends of PCL and multifunctional acrylate monomers were also investigated to improve radiation cross-linking efficiency [33].

Nagata et al. [34,35] studied the biodegradability and shape-memory properties of a synthesized multiblock copolymer derived from PCL, 4,4-(adipoyl di-oxy)dicinnamic acid, and poly(ethylene glycol) (PEG). Petrova et al. [36] and Cohn et al. [37] reported that PCL/PEG copolymers can exhibit an exceptional combination of properties, such as hydrophilicity, permeability, and degradability. A less tight network was observed from the swelling study in cases of increased PEG content. Lendlein et al. [38] investigated the mechanical and SMP properties of PCL-based dimethacrylates of different molecular weights, ranging from 1500 to 10,000 g/mol. The maximum Young’s modulus of 3.01 MPa and recovery ratio of 95.5% were obtained from PCL of 3500 g/mol and 10,000 g/mol, respectively. Ping et al. [39] stretched PCL-based polyurethane (PCLU) at 10–15 °C lower than T_m_ and allowed it to recover at a lower temperature. They concluded that the lowest recovery temperature (LRT) mostly depends on the molecular weight rather than the percentage of hard segments. However, the main limitation of PCL-based biodegradable SMPs is that the product strength after shape recovery is insufficient for some practical applications [40]. To solve this problem, Chen et al. synthesized a series of thermosetting SMPUs using diethanolamine as the chemical cross-linker [41]. Tan et al. utilized a post-cross-linking method to endow an SMPU containing an elastic cross-linking structure. Luo et al. found that using a small amount of planar cross-linker to cross-link SMPUs slightly can cause it to exhibit excellent thermal and shape-memory properties [42].

Previously, our group successfully prepared a shape-memory PET/PCL copolyester that exhibits acceptable process temperature, tunable and reversible shape-memory behavior, and good spinnability [8]. However, the mechanical properties still need improvement. NDC, also known as naphthalene dicarboxylate, is a co-monomer that has been used in the production of copolyesters such as PET/PEN. It is known to improve the thermal and mechanical properties of the resulting material, making it more resistant to deformation and higher temperatures. Several studies on PET/PEN copolymers have shown that the addition of NDC can lead to significant improvements in the properties of the resulting copolymer. For example, Pavel’s [43] modeling work demonstrated that NDC can enhance the stiffness and thermal stability of PET/PEN copolymers, while Lee’s work [44] on the synthesis of PET/PEN copolymers using NDC as a co-monomer showed improvements in tensile strength and glass transition temperature. To our knowledge, there are no reports that have employed planar crosslinkers in the synthesis of polyesters to further investigate their shape-memory properties. Based on our previous study, we employ planar (trimesic acid) and non-planar (glycerol) cross-linkers to synthesize cross-linking polyesters. Meanwhile, a rigid PEN structure is also incorporated through polymerization.

## 2. Materials and Methods

### 2.1. Materials

The following chemicals were used in this experiment: terephthalic acid (TPA; 99.9%; Oriental Petrochemical, Taoyuan, Taiwan), ethylene glycol (EG; 99.9%; Emperor Chemical, Taipei, Taiwan), glycerol (GC; 99.7%; Emperor Chemical, Taipei, Taiwan), benzene-1,3,5-tricarboxylic acid (BTC; 95%; Aldrich, St Louis, MO, USA), dimethyl 2,6-naphthalenedicarboxylate (NDC; 98%; Aldrich), titanium(IV) butoxide (TBT; 97%; Aldrich), trifluoroacetic acid-d (TFA-d; 99.5%; Aldrich), phenol (97%; Aencore Chemical; Surrey Hills, Australia), toluene (99%; Aldrich), lipase from porcine pancreas (15–35 units per mg; Aldrich), phosphate-buffered saline (powder, pH 7.4, for preparing 1 L solutions; Aldrich), 1,1,2,2-tetrachloroethane (97%; Showa Chemical Industry; Tokyo, Japan), and PCL-diol (Mw = 3000 g/mol) (Daicel, Tokyo, Japan). The PCL (Mw = 50,000 g/mol) was provided by TSM Smart Materials (Taoyuan, Taiwan). All reagents were used as received without further purification.

### 2.2. Synthesis of PET and PET/PEN/PCL Copolyesters

**Scheme 1:**
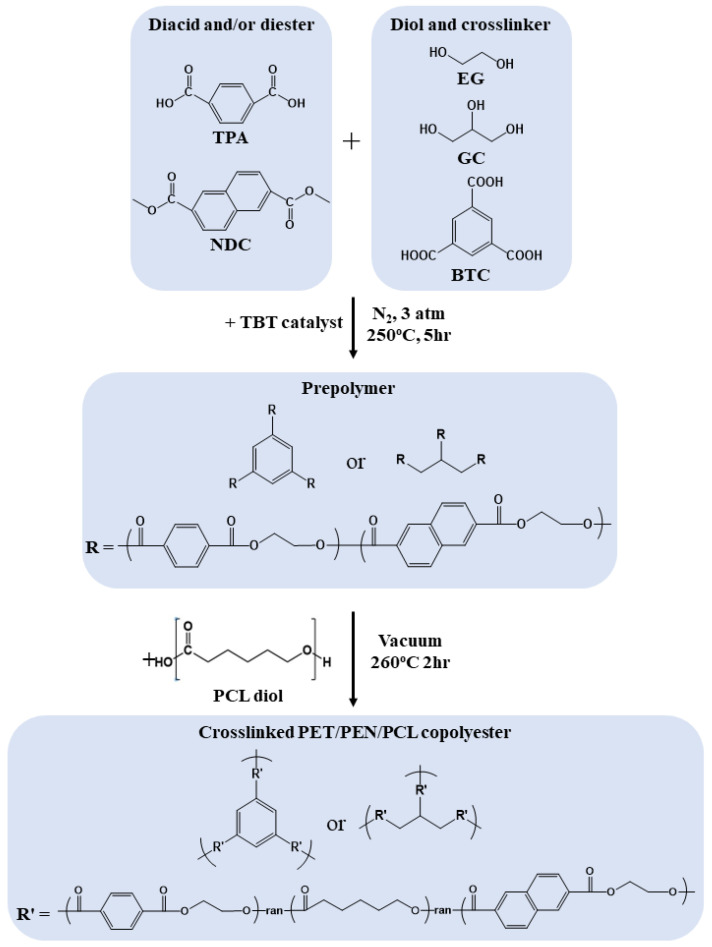
Synthesis route of cross-linked PET/PEN/PCL copolyesters.

The synthesis process is presented in Scheme 1. For the synthesis of PET homopolymer and PET/PEN/PCL copolyesters with or without a cross-linker, a one-pot polycondensation method was adopted. A stainless-steel 2 L reactor equipped with a mechanical stirrer, nitrogen inlet and outlet, temperature sensor, distillation column, and vacuum system was used.

In brief, first, TPA, EG, cross-linker (GC or BTC), and TBT catalysts were charged in the reactor. An esterification reaction between TPA, EG, and cross-linker was performed by stirring at 80 rpm at 250 °C under a nitrogen flow at a pressure of 3 bar. The diester-to-diol molar ratio was set at 1:1.2, and the amount of TBT was retained at 400 ppm based on the theoretical amount of oligo(ethylene terephthalate). The degree of esterification was determined by measuring the amount of cool-condensed H_2_O and CH_3_OH, and the endpoint of the esterification reaction was selected as an esterification degree of more than 90%.

For the polycondensation reaction, the PCL-diol was added to the reactor under a nitrogen atmosphere. After the reactants were moderately mixed to form a homogeneous melt, the polycondensation reaction was conducted by gradually increasing the temperature from 250 to 260 °C and decreasing the pressure from 760 to less than 1 Torr over a 30 min period. The temperature and vacuum were then maintained to remove excess diols and by-products until the end of the reaction. The reaction lasted approximately 1–3 h. The exact endpoint of the polycondensation reaction was selected as the maximum value of stirring torque.

Finally, the melted polymer was plunged into ice water, pelletized using a granulator into approximately 2 mm pellets, dried in a vacuum oven at 105 °C for 4 h, and stored in an aluminum foil-sealed bag with a desiccant to keep the pellets dry until further analysis and processing.

A series of PET/PEN/PCL copolyesters with different cross-linkers was named 30CXY or 30CNXY, where 30C means the total weight ratios of PCL content. X and Y denote the type of cross-linker (G = GC, B = BTC) and the molar ratio of that cross-linker to TPA + NDC, respectively. N denotes the sample whose molar ratio of TPA was 1/3 replaced by NDC.

### 2.3. Nuclear Magnetic Resonance Spectroscopic Analysis

A Bruker Avance III HD-600 MHz nuclear magnetic resonance (NMR) spectrometer (Bruker, Germany) was used to identify the synthesized copolyesters. Briefly, 5–10 mg of polymer was fully dissolved via ultrasonic shock in 1 mL of TFA-d in a 10-mL vial and then transferred to 5 mm NMR spectroscopic analysis (^1^H-NMR) sample tubes. All experiments were performed at 25 °C, with 64 scans recorded.

### 2.4. Fourier Transform Infrared Spectroscopy

For Fourier transform infrared spectroscopy (FT-IR), the samples were prepared as thin films of approximately 300 μm using hot-pressure mechanics at a temperature approximately 20–30 °C above the melting temperature. For copolyesters without a melting temperature, a temperature of 200 °C was used. The FT-IR spectra ranging from 4000 to 650 cm^−1^ were recorded using a PerkinElmer Spectrum One spectrometer (Waltham, MA, USA) in attenuated total reflection mode at a resolution of 4 cm^−1^, with 16 scans.

### 2.5. Intrinsic Viscosity

The intrinsic viscosity [*η*] of the polymers was measured using the ASTM D4603 method. The polymers were dissolved in a mixture of phenol and 1,1,2,2-tetrachloroethane at a weight ratio of 60:40 at a concentration of 0.5 g/dL. The intrinsic viscosity measurement was performed using a Cannon Ubbelohde Type 1B Viscometer at 30 ± 0.05 °C. The [*η*] values of the copolyesters were calculated using the Billmeyer relationship [45]:

(1) $$\left[\eta \right]=0.25\left[\eta_{r}-1+3\ln\left(\eta_{r}\right)\right]$$

where $\eta_{r}$ is the relative viscosity.

### 2.6. Differential Scanning Calorimetry

Differential scanning calorimetry (DSC) measurements were performed using a differential scanning calorimeter system (DSC 8000, PerkinElmer, Waltham, MA, USA). Samples were cut from the pellets and dried in a vacuum oven at 105 °C for 1 h. Each sample had a mass of 4–6 mg and was sealed in an aluminum pan. The measurements were conducted over a temperature range of 0 to 280 °C, at a heating and cooling rate of 10 °C/min under a nitrogen flow rate of 20 mL/min. The conventional triple-cycle heating–cooling–heating process was employed. The temperature was maintained at 280 °C for 3 min in the first heating stage to remove the thermal history. The melting temperature (T_m_), crystallization temperature (T_c_), and cold crystallization temperature (T_cc_), and the corresponding enthalpies ΔH_m_, ΔH_c_, and ΔH_cc_, were determined as the peak temperature and integral values of that peak, respectively.

### 2.7. Dynamic Mechanical Analysis

DMA (EXSTAR 6100 DMS, Seiko Instruments Inc., Chiba, Japan) was used to measure the viscoelastic properties of the specimens. Approximately 1 mm-thick plates were obtained using a hot-press method similar to that used in the FT-IR section. The plates were then cut into narrow strips of 20 × 5 mm^2^. The samples were analyzed in tension mode with a minimum force of 100 mN over the temperature range of −75 to 200 °C, at a rate of 5 °C/min and a fixed frequency of 1 Hz.

### 2.8. Thermogravimetric Analysis

Thermogravimetric (TGA) analyses were performed using a thermogravimetric analyzer (STA 7200, HITACHI, Tokyo, Japan). 5–6 mg was accurately weighed and placed in a ceramic furnace, and the test was conducted under a nitrogen atmosphere at a flow rate of 100 mL/min, with heating from 30 to 600 °C at a rate of 10 °C/min. The characteristic onset of the degradation temperature was determined from the TGA curve at 5% weight loss (T_d-5%_).

### 2.9. Equilibrium Swelling Analysis

To determine the degree of cross-linking of the samples, an equilibrium swelling method was used. Each sample, weighing approximately 1 g, was first soaked in boiling toluene for 8 h using a Soxhlet extractor and then allowed to equilibrate with toluene at 25 °C for 24 h. The swollen polymer’s mass (m) was measured, and the specimen was subsequently dried in a vacuum and weighed again to obtain the mass of the dry polymer ($m_{p}$). The swelling ratio was then calculated using the following formula:

(2) $$Q=\frac{m_{p}/\rho_{p}+(m-m_{p})/\rho_{s}}{m_{p}/\rho_{p}}$$

where $\rho_{s}$ and $\rho_{p}$ are the densities of toluene and the polymer. The density of cross-links in the polymer was calculated using the Flory-Rehner equation [46]:

(3) $$N=\frac{-(\ln\left(1-v_{p}\right)+v_{p}+\chi v_{p}^{2}}{V_{s}(v_{p}^{1/3}-\frac{v_{p}}{2})}$$

where $v_{p}$= 1/Q is the volume fraction of polymer in swollen state, $V_{s}$ = 1.06 × 10^−4^ m^3^/mol is the molar volume of the solvent (toluene), and χ is the Flory-Huggins interaction parameter. The value of χ can be estimated according to the equation:

(4) $$\chi =\frac{{\left(\delta_{s}-\delta_{p}\right)}^{2}V_{s}}{RT}$$

The Hildebrand solubility parameter at 25 °C for the toluene ($\delta_{s}$) is 18.2 MPa^1/2^. According to the values of Hildebrand solubility parameters at 25 °C for PCL = 19.5 MPa^1/2^, PET = 21 Mpa^1/2^, and PEN = 23 Mpa^1/2^, based on the molar fractions of PET, PEN, and PCL in the copolymer, the Hildebrand solubility parameter of the copolymer ($\delta_{p}$) can be calculated by weighted average [47].

### 2.10. Wide-Angle X-ray Diffraction

For wide-angle X-ray diffraction analysis, approximately 0.1 × 10 × 10 mm^3^ specimens were obtained by hot pressing. The X-ray diffraction patterns of the film samples were recorded using a X-ray powder diffraction instrument (X Pert^3^, Malvern, Worcestershire, UK) equipped with a CuKα radiation source (λ = 0.154 nm) and were then verified in the 2θ range of 10°−40° with a scanning speed of 0.2°/min.

### 2.11. Tensile Test

For the tensile tests, dumbbell-shaped specimens were prepared by injection molding (Thermo Fisher Scientific, Minijet Pro, Waltham, MA, USA). The polymer was fed into a feeder and preheated for 5 min. Once the polymer had completely melted, it was injected into the dumbbell-shaped mold at an injection pressure of 350 bar, and a pressure of 250 bar was maintained for 30 s to remove any bubbles. The preheating and injection temperatures were the same as those used for hot pressing, and the mold temperature was half that of the injection temperature. Tensile properties were investigated using a Come-tech QC-508M2F tensile testing machine, and the dumbbell size and conditioning followed ASTM D638 type IV standards. Each sample was analyzed at a crosshead speed of 20 mm/min. Young’s modulus, yielding strength, elongation at break, and tensile strength were evaluated using the stress-strain data. The average values were obtained from five specimens.

### 2.12. Enzymatic Degradation

For the degradation experiments, the dried polymer films (20 × 50 × 0.1 mm^3^) were gently shaked in a solution of lipase/0.1 M PBS (pH = 7.4) (50 mg/50 mL) at 37 °C. These films were taken out and refreshed thoroughly with distilled water, and then dried in a vacuum oven at 40 °C until weight balanced (~16 h) every week. The degree of degradation was evaluated by the weight loss calculated as following:

(5) $$Weight\;loss\left(\%\right)=\frac{W_{0}-W_{t}}{W_{0}}\times 100\%$$

where W_0_ is the weight of the dried films before degradation and W_t_ is the weight of the dried films at degradation time t. A higher weight loss (%) implies better degradability of the films.

### 2.13. Shape-Memory Properties

The film specimens, with a sample size of 10 × 10 × 0.1 mm^3^, were employed to evaluate shape-memory behaviors using a DMA (EXSTAR 6100 DMS, Seiko Instruments Inc., Chiba, Japan) under SS-Control mode. Each specimen was maintained at its pull temperature and stretched at a force rate of 600 mN/min for 4 min. It was then cooled to its fixing temperature at a cooling rate of 23 °C/min while maintaining the force. Once the fixing temperature was reached, the force was removed from the specimen, it was left for 5 min to ensure stress release, and the temporary strain (ε_temp_) was measured. The fixed specimen was then reheated to its recovery temperature at a rate of 3 °C/min. During the reheating process, the recovery behavior and the strain of the recovered shape (ε_rec_) were recorded. The shape fixation ratio (R_f_) and shape recovery ratio (R_r_) were calculated as follows [10]:

(6) $$R_{f}(N)=\frac{\varepsilon_{temp\;}}{\varepsilon_{load}}\times 100\%$$

(7) $$R_{r}(N)=\frac{\varepsilon_{temp}-\varepsilon_{rec}}{\varepsilon_{temp}-\varepsilon_{int}}\times 100\%$$

where ε_load_ is the maximum strain applied during programming, ε_int_ is equal to the initial strain of each cycle, and N is the number of shape recovery cycles. The pull temperature and the recovery temperature were set to the same temperature, with 70 °C used for the PET/PCL series and 50 °C used for the PET/PEN/PCL series. For the fixing temperature, 0 °C and −20 °C were chosen for the PET/PCL series and PET/PEN/PCL series, respectively.

## 3. Results and Discussion

### 3.1. Synthesis and Structure Characterization of the Copolyesters

**Figure 1:**
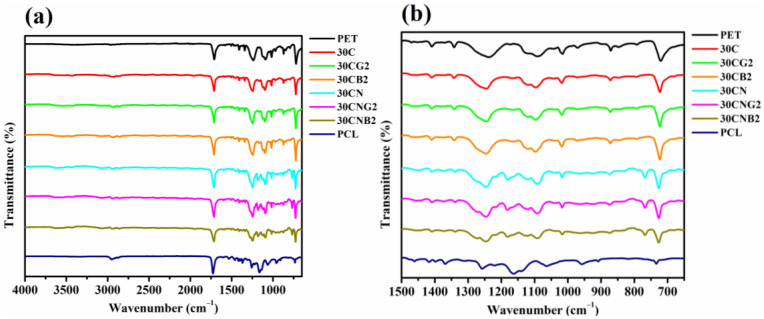
Infrared spectra of the PET, PCL, and PET/PEN/PCL with wavenumber (**a**) from 650 to 4000 and (**b**) 650 to 1500 cm^−1^.

**Figure 2:**
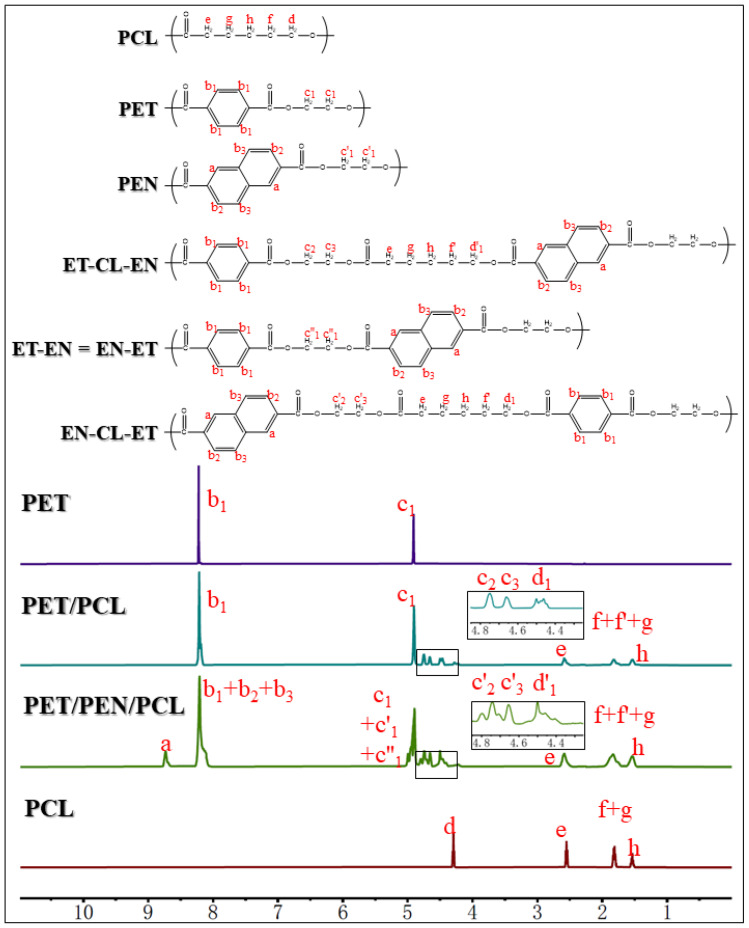
^1^H-NMR spectra of the PET/PEN/PCL and the signal assignment of possible molecular segment arrangements.

The composition of the PET/PEN/PCL copolymers is listed in Table 1, and their chemical structures were confirmed using FT-IR (Figure 1 and Table 2) and ^1^H-NMR (Figure 2 and Table 3). In the FT-IR spectra, the absorption bands at 2967 and 2963 cm^−1^ were the signals of symmetrical and asymmetrical methylene stretching vibrations, respectively. These signals increased with the increase of PCL content due to the higher methylene content. For samples containing benzene and naphthalene rings, the corresponding peaks on the spectrum were at 1336, 1179, 1120, 871, and 764 cm^−1^. All polyesters and copolyesters showed the characteristic peak of carbonyl stretch at 1720 cm^−1^. Thus, the signals presented in the FT-IR spectra indicated that the PET/PEN/PCL copolymers had been synthesized successfully.

To further investigate the chemical structure of the PET/PEN/PCL copolymer and verify that copolymerization had occurred, ^1^H-NMR analysis of the PET/PEN/PCL spectra was conducted. The signal assignments for the possible segment units of the PET/PEN/PCL copolymer are listed in Figure 2. These spectra were compared with those of neat PET and PCL. The characteristic peaks of neat PET appeared at δ = 8.1–8.3 (b) and δ = 4.9 (c), while those appearing at δ = 4.25 (d), δ = 2.5 (e), δ = 1.8 (f, g), and δ = 1.5 (h) belong to neat PCL. The upfield shift of peak c and downfield shift of d located between δ = 4.4–4.8 (c_2_, c′_2_, c_3_, c′_3_, d_1_, d′_1_) indicated that covalent bonding between PET/PEN and PCL had occurred. The molar ratio of the components of each copolyester can be obtained from the following equations:

(8) $$N_{ET}=\left\{\begin{array}{l} \frac{A_{b}}{4}\\ \frac{A_{b}}{4}\times \frac{2}{3}(when NDC incorporated) \end{array}\right.$$

(9) $$N_{EN}=\frac{A_{a}}{2}$$

(10) $$N_{EG}=\frac{A_{c+c^{\prime }1+c^{\prime \prime }1}}{4}$$

(11) $$N_{CL}=\frac{A_{h}}{2}$$

where N_ET_, N_EN_, N_EG_, and N_CL_ represent the molar ratios of TPA, NDC, EG, and CL repeating units, respectively. A_x_ represents the integral values of the corresponding peak x. The output molar ratios are shown in Table 1. It can be seen that the ratio of TPA to NDC in the product is almost the same as the theoretical input value, while the molar ratio of CL is between 0.30 and 0.36, which is lower than the expected value of 0.51 to 0.55. This may be due to the poor thermal stability of PCL-diol, which was susceptible to thermal degradation during the condensation reaction at 280 °C. Part of the PCL-diol is subject to chain scission and carried away by the vacuum system along with the by-products. The decrease in the output EG ratio confirms that some of the ET-EN segments were replaced by ET-CL-EN or EN-CL-ET segments, indicating that copolymerization of PCL indeed occurred.

The values of the average repeating units of the hard segments, ET + EN (m + l), in each macromolecule can be obtained from the change in the peak area assigned to the EG unit between two benzene or naphthalene rings, X_EG′_, which is assumed to be equal to that of peak b for neat PET or PEN. X_EG′_ is calculated using Equation (9):

(12) $$X_{{EG}^{\prime }}=\frac{N_{ET}+N_{EN}-N_{EG}}{N_{ET}+N_{EN}}$$

The chemical environmental difference of EG in the copolymer compared to the PET or PEN homopolymer results in a loss of N_EG_. The increase in X_EG′_ indicates a decrease in the hard segment length, which can be calculated as follows:

(13) $$m+l=\frac{1}{X_{{EG}^{\prime }}}$$

The average repeating units of the soft CL segments, n, can be calculated by the molar fraction of the CL units, X_CL_, in the copolyesters using the following relationship:

(14) $$X_{CL}=\frac{N_{CL}}{N_{ET}+N_{EN}+N_{CL}}$$

(15) $$n=\frac{(m+l)\times X_{CL}}{1-X_{CL}}$$

The distribution of soft and hard segments in the polymer chains can be evaluated by degrees of randomness:

(16) $$R=\frac{1}{n}+\frac{1}{m+l}$$

The results show that samples without the naphthalene component (30C, 30CG2, and 30CB2) have higher X_EG′_ (0.34–0.36), which suggests that more ethylene adjacent to aromatic rings is replaced by the CL segment, although their theoretical input molar ratios of PCL-diol are slightly lower than those of the sample with the naphthalene component (30CN, 30CNG2, and 30CNB2). Nevertheless, for the 30CN series samples, the segment length of both the soft and hard segments were slightly longer than those of the 30C series samples, and their degrees of randomness were closer to 1. This indicates that the 30CN series samples have longer chain segments and the distribution tends to be random [48]. However, from the analysis of FT-IR and ^1^H-NMR, we cannot clearly see the influence of the cross-linking agent because of the low amount.

### 3.2. Thermal Properties of the Copolyesters

For thermotropic shape-memory polymers, the states of crystallization, cross-linking, and chain entanglement have a significant impact on their shape-memory properties and applications. In this section, DSC, DMA, and TGA were utilized to study the thermal behavior of PET/PEN/PCL cross-linked by GC or BTC.

**Figure 3:**
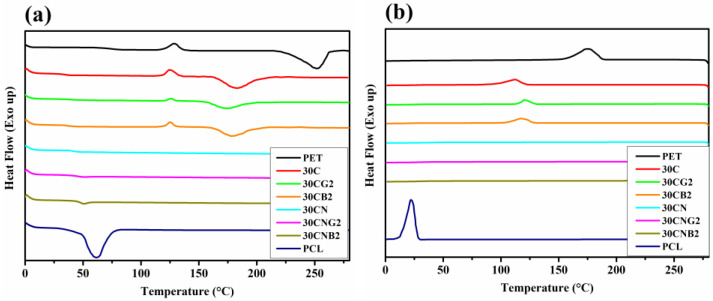

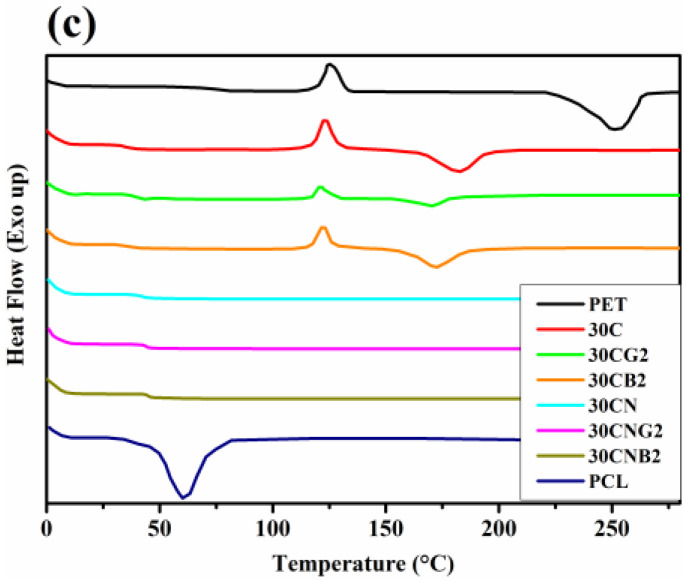
DSC thermograms of (**a**) first heating, (**b**) first cooling, and (**c**) second heating scans of the PET, PCL, and PET/PEN/PCL.

In Figure 3, the triple-cycle heating–cooling–heating DSC curve reveals that the thermal behavior of PET/PEN/PCL is dependent on the composition and process conditions. The calorimetric parameters, T_g_ measured through DMA, and thermogravimetric parameters of PET/PEN/PCLs are provided in Table 4. As shown in Figure 3c, the introduction of the CL segment disrupts the chain’s regularity of PET, which leads to a decrease in the melting point from 250.6 to 182.3 °C. The further introduction of the EN segment leads to a more serious disruption of regularity, resulting in the disappearance of T_m_ on the DSC thermogram. The PET homopolymer had the highest T_g_ value because it possessed the highest ratio of benzene rings, which suppressed chain mobility. Due to the superior molecular mobility of the aliphatic CL segment compared to that of the aromatic ET segment, all samples containing a molar ratio of about 30/100 of CL/aromatic exhibited lower T_g_ than PET. At a similar CL molar ratio, the samples with a naphthalene ring partially substituted for the benzene ring possessed a higher T_g_, indicating the more rigid structure of naphthalene than benzene. The recrystallization peaks for PET, 30C, 30CG2, and 30CB2 were observed in both the first heating curve (Figure 3a) and the second heating curve (Figure 3c). The higher T_m_, T_cc_, and ∆H_m_ and lower ∆H_cc_ indicate that the crystallinity was enhanced through annealing before the first heating curve. The effect of different cross-linkers on the thermal behavior was also observed. Cross-linking with BTC or GC for 30C and 30CN, respectively, enhanced the T_g_ values from 34.2 to 38.9 °C and from 42.5 to 46.8, respectively. Here, BTC and GC caused different trends of improvement in T_g_. For 30C, the improvement effect of GC is better than that of BTC, but the opposite is true for 30CN. This may be due to the fact that the cross-linking efficiency of GC for 30C is better than that of BTC, but not for 30CN. A higher cross-linking efficiency will result in a higher cross-linking degree, which will limit the mobility of molecular chains, thus resulting in a more significant improvement of T_g_ in 30CG2. The higher cross-linking density could also hinder the packing of crystalline structure, leading to the lower T_m_ and ∆H_m_ for 30CG2 compared to 30CB2. Besides the reduction of crystallinity, in the cooling curve (Figure 3b), the increase in Tc for 30C after cross-linking suggests that the introduction of partial cross-linked nodes acted as nucleating agents to improve the rate of crystallization.

**Figure 4:**
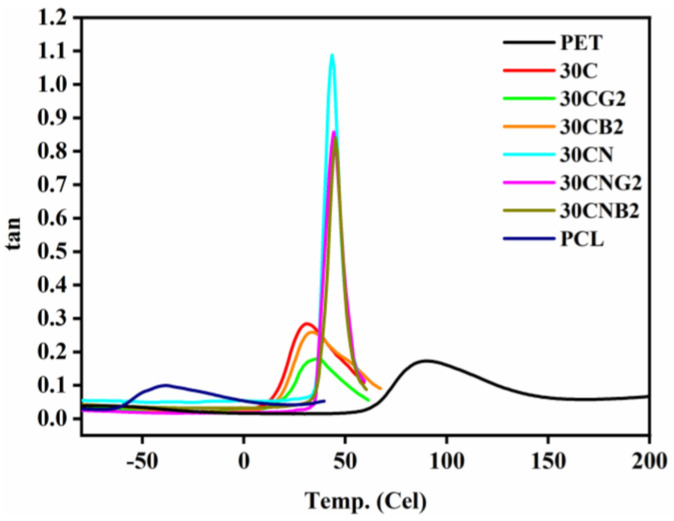
The loss tangent of the PET, PCL, and PET/PEN/PCLs determined by DMA.

Figure 4 displays the damping factor (tanδ) of PET, PCL, and PET/PEN/PCLs cross-linked with different cross-linkers as a function of temperature at 1 Hz. As the tanδ is calculated by dividing the loss modulus by the storage modulus, it is used to assess the viscoelasticity of polymer materials. Comparing the tanδ of 30CG2 versus 30CB2 and 30CNG2 versus 30CNB2, the lower tanδ of 30CG2 and the similar tanδ of 30CG2 and 30CNB2 indicate a higher cross-linking density of 30CG2 versus 30CB2 and a similar cross-linking density of 30CNG2 versus 30CNB2. As discussed in the previous paragraph, the T_g_ value could be affected by both the rigidity of the structural unit and the degree of cross-linking. Based on the tanδ results, the higher cross-linking density of 30CG2 leads to different trends in T_g_ for 30C and 30CN cross-linked by GC or BTC. Despite the greater rigidity of BTC compared to GC, the trends of T_g_ determined by DMA, which were defined as the peak of the tanδ curve, are consistent with the DSC results. Notably, these copolyesters containing 30 phr of PCL had T_g_ ranging from 34.2 °C to 46.8 °C, which is close to body temperature and could be adjusted by different hard segments and cross-linkers, indicating their potential for use in personalized thermoregulation.

**Figure 5:**
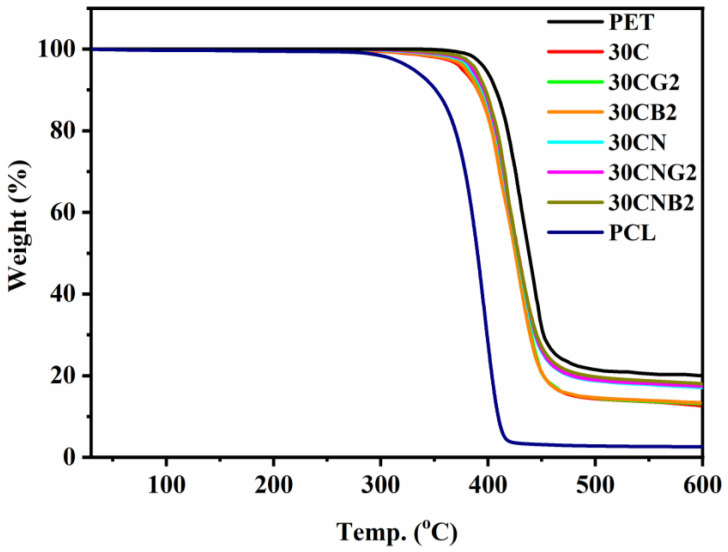
Weight loss as a function of temperature for PET/PEN/PCL copolyesters.

The thermal stability of polymers is a key factor affecting their application fields. Poor thermal decomposition temperature (T_d_) can limit their use. The TGA thermograms in Figure 5 demonstrate that incorporating the aliphatic CL segment decreased the initial degradation temperature compared to that of a PET homopolymer. Table 4 summarizes the results of Figure 5 and reveals that the T_d-5%_ values of the PET/PCLs and PET/PEN/PCLs were in the range of 376.1–381.0 °C and 383.4–388.2 °C, respectively, which is lower than that (397.9 °C) of a PET homopolymer. In the early stage of degradation, the cross-linking density has a more pronounced impact on T_d-5%_ because the existence of those cross-linking points enables the polymer to maintain a moderate molecular weight. It takes more energy and time to cleave the polymer into smaller molecules and then evaporate. In the later stage of degradation, most of those aliphatic cross-linking points have been broken. At this time, the impact of structural units on the residual weight is more significant, resulting in a higher T_d-5%_ and lower char of 30CG2 compared to 30CB2. However, all PET/PCLs and PET/PEN/PCLs exhibit good thermal stability under TGA analysis, which is suitable for most polymer processing methods such as injection molding and melt spinning.

### 3.3. Mechanical Performance of the Copolyesters

**Figure 6:**
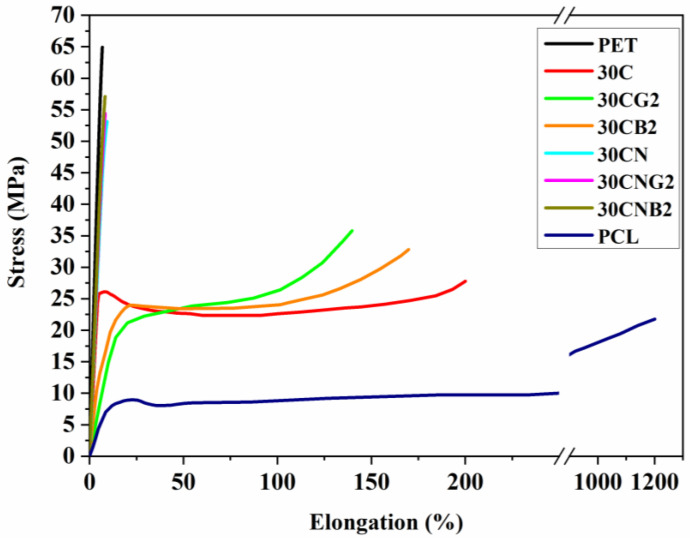
Stress–strain curves for PET, PCL, and the PET/PEN/PCLs.

The tensile properties of the polymer were determined, and the results are presented in Table 5 and Figure 6. The tensile strength and elongation at break of PET/PCLs and PET/PEN/PCLs ranged between those of PET homopolymer and PCL homopolymer, depending on the composition and type of cross-linker. First, when 30 phr of PCL-diol is introduced into PET (30C), the elongation at break of PET increases from 6.8% to 200.1%, and the stress–strain behavior changes from brittle to ductile. On the other hand, the introduction of PCL-diol to PET improves the strength of PCL from 21.8 MPa to 27.8 MPa, and the Young’s modulus is greatly promoted from 89.3 to 556.0 MPa. However, replacing 1/3 of the ET segment with the EN segment of 30C, which is 30CN, causes the stress–strain behavior to be similar to PET again. This enables 30CN to maintain a high tensile strength of 53.2 MPa compared with 27.8 MPa of 30C under the same CL segment ratio.

The different effects of GC and BTC on the mechanical properties of 30C and 30CN were observed. In all cases, cross-linking with GC or BTC led to a decrease in elongation and an increase in tensile stress. Among them, the effect of 30CG2 was the most dominant, indicating that the composition exhibited the highest degree of cross-linking, the movement of molecules was restrained to the greatest extent, and the energy required to destroy the specimen was also greatly improved. The opposite effect on the Young’s modulus of 30C and 30CN with GC and BTC was found. This may be caused by the decrease in the crystallinity of the 30C series. The increasing crystalline defects make the crystalline structure split in the early stage of the tensile test more easily, which reduced the Young’s modulus of 30CG2 and 30CB2 from 556.0 to 151.2 and 257.9, respectively. On the contrary, the absence of crystalline structure in the 30CN series makes it unaffected. As a result, the Young’s modulus of 30CNG2 and 30CNB2 improved from 675.4 to 763.5 and 773.1, respectively, owing to the rigid nature of the aromatic structural BTC.

The intrinsic viscosity of a polymer is affected by its structural unit, molecular weight, and degree of cross-linking. Generally, a polymer with a soft structural unit is easier to stretch when dissolved in a solvent, resulting in a higher viscosity. When comparing polymers of the same structure, a higher molecular weight or a higher degree of cross-linking will hinder fluid flow, leading to an increase in intrinsic viscosity. The intrinsic viscosity results presented in Table 5 show that the cross-linking degree of 30CG2 is significantly higher than that of 30CB2, resulting in higher strength, lower elongation, lower Young’s modulus, and lower yield stress.

**Figure 7:**
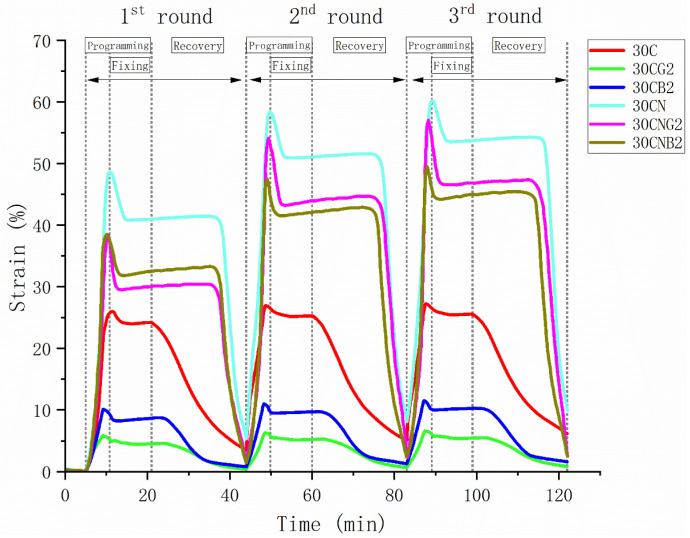
2D diagram of SMP behavior of PET/PEN/PCLs.

**Figure 8:**
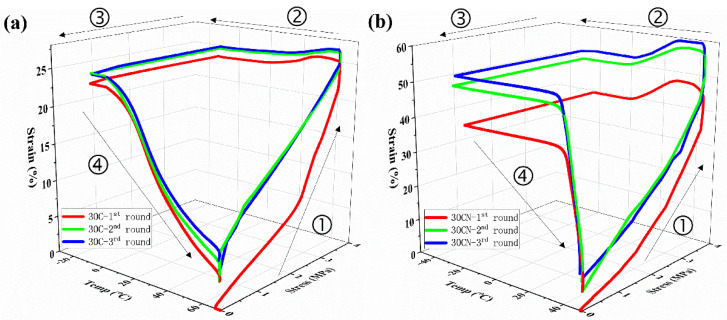
3D diagram of SMP behavior in the following steps; (1) stretching at fixed pulling temperature, (2) cooling at constant stress, (3) releasing stress at fixed fixing temperature, and (4) heating without applying external stress of (**a**) 30C (PET/PCL) and (**b**) 30CN (PET/PEN/PCL).

**Figure 9:**
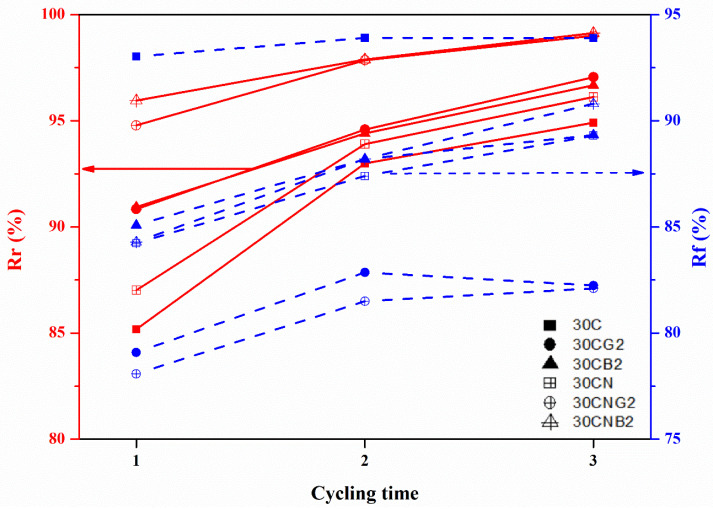
The shape recovery ratio (R_r_) and shape fixation ratio (R_f_) of PET/PEN/PCLs.

The shape-memory behavior of PET/PEN/PCLs is depicted in Figure 7 and Figure 8. Three rounds of shape-memory tests were conducted on the samples, and their R_r_ and R_f_ values were calculated and summarized in Figure 9 and Table 6. Firstly, it is evident from Figure 7 and Figure 8 that the shape-memory behavior of the first round significantly differed from the second and third rounds in all cases. This is due to the physical chain entanglements or intermolecular interactions in the amorphous region being easily disrupted during the initial deformation. However, with an increase in the number of deformations, the molecular chains in the amorphous region become oriented, resulting in a more stable state; therefore, more consistent shape-memory performance is observed in the second and third rounds.

Comparing the plots of 30C and 30CN, the experimental results indicate that under the same maximum stress (4 MPa), 30CN is more susceptible to stretching than 30C, even at lower processing temperatures. The ε_load_ increased from 26 to 27% for 30C and from 49 to 60% for 30CN, respectively. Due to the lack of crystallinity in 30CN, there is no fixed crystalline phase to maintain its shape at temperatures above T_g_. This also leads to a decrease in the R_f_ of 30CN compared to 30C, from 93% to 84.2%. However, in terms of R_r_, there is little difference between 30C and 30CN, which are 85–95% and 87–96%, respectively, indicating that the addition of a naphthalene ring has little effect on the recovery rate of the PET/PCL system.

Furthermore, the effects of using GC and BTC as cross-linkers on the shape-memory behavior of 30C and 30CN were compared. As expected, the introduction of cross-linking agents increased the elasticity of the polymer, resulting in lower ε_temp_ and higher R_r_ for all samples. However, GC and BTC had different effects on the R_f_ of 30C and 30CN. For the 30C series, the addition of cross-linking agents reduced the Rf from 93.9% to 82.2%, whereas for the 30CN series, the addition of BTC increased the R_f_. This phenomenon can be explained by two aspects. As mentioned above, the morphology of the stationary phase is the main factor affecting R_f_. For 30C, the crystalline phase contributes to the main stationary phase; therefore, the addition of cross-linking agents leads to more defects in the crystalline region, resulting in a decrease in R_f_. Moreover, since GC breaks the crystalline region to a greater extent, a more significant decrease in Rf was observed in 30CG2. In contrast, as amorphous polymers, the 30CN series exhibits a lower fixed rate than that of the 30C series. When the temperature is above T_g_, the shape of these amorphous polymers can only be maintained by the physical chain entanglements or intermolecular interactions in the amorphous regions. Under such circumstances, the introduction of the symmetric planar cross-linker, BTC, can provide some cross-linking nodes for the molecular chains. These rigid cross-linking nodes may reduce the mobility of the molecular chains at moderate temperatures above T_g_, resulting in an improvement in R_f_ in 30CNB2. Finally, comparing the recovery curves of the 30C and 30CN series, it was found that the recovery behavior of 30CN occurred over a smaller temperature range. This shows that 30CNs, as amorphous polymers, have higher temperature sensitivity than 30Cs, the crystalline polymers, and are more suitable for use in shape-memory products where precise temperature control is necessary.

### 3.4. Controllable Degradation of Cross-Linked Copolyesters

**Figure 10:**
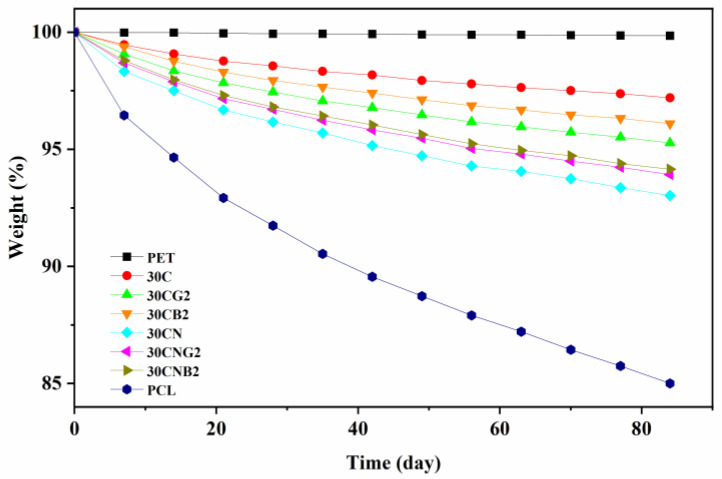
Remaining weight curves of PET, PCL and the PET/PEN/PCLs during emzymatic degradation.

Implantation for in vivo applications is one of the primary uses of shape-memory polyester, and for such applications, the biocompatibility and biodegradability of materials are of concern. Jana et al. [49] have proven the non-cytotoxicity of pristine polyethylene naphthalate, and the well-known biocompatibility of PET and PCL indicates that the copolyester consisting of PET, PCL, and PEN can be considered biocompatible. Lee et al. [50] incorporated dihydroxyterephthalate (DHTE) as a “Trojan Horse” counit into PET for facile chemical recycling. Based on an investigation of various reaction parameters, such as catalyst concentration, hydrolysis time/temperature, and DHTE loading, it was found that hydrolytic degradation of PET/DHTE copolymers using dilute metal catalytic aqueous solutions can proceed easily under much milder conditions compared to traditional PET hydrolysis, with no emission of toxic substances. Here, the biodegradability of PET/PEN/PCL copolyesters was investigated. Figure 10 displays the enzymatic degradation behavior of PET, PCL, and PET/PEN/PCL copolyesters with various compositions and cross-linkers over 84 days, using PBS to simulate the environment inside the human body.

The results of the experiment indicate that PET exhibited minimal degradation under the tested conditions, whereas PCL degraded at a rate of 15%. The degradation behavior of the 30Cs and 30CNs was found to be intermediate between that of PET and PCL. By referring to Table 1, it was determined that the weight percentages of the CL segment in the 30C and 30CN copolyesters were 15.1% and 17.6%, respectively. Assuming that the degradation rates of PET and PEN were close to zero, and that the degradation of the copolymers was entirely attributable to the CL component, the theoretical degradation rates of the 30C and 30CN copolyesters were estimated to be 2.27% and 2.64%, respectively. This was calculated by multiplying the degradation rate of PCL in Figure 10 by the weight percentage of the CL segment in each copolyester. However, the actual degradation rates of all copolymers ranged from 2.8% to 7.0%, which is significantly higher than the theoretical values.

**Figure 11:**
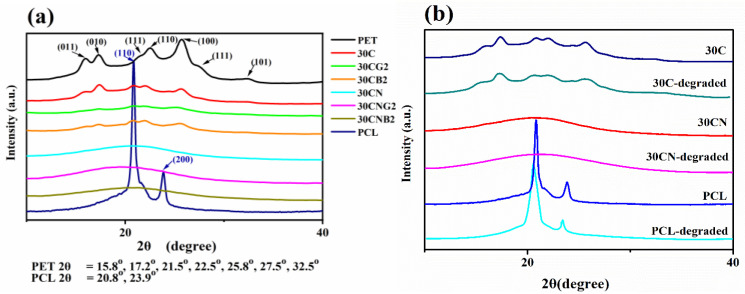
XRD patterns of (**a**) pristine PET, PCL, and PET/PEN/PCL copolyesters cross-linked with GC and BTC and (**b**) 30C, 30CN, and PCL before and after degradation.

To gain further insight into the relationship between degradation behavior and crystalline morphology and cross-linking, wide-angle diffraction was utilized. The XRD pattern of PET, PCL, and the copolyesters is presented in Figure 11a. PET displays seven characteristic peaks at 2θ = 15.8°, 17.2°, 21.5°, 22.5°, 25.8°, 27.5°, and 32.5°, whereas PCL exhibits two characteristic peaks at 2θ = 20.8° and 23.9°. Upon the introduction of PCL-diol and cross-linking agent, the intensity of the characteristic peaks associated with PET and PCL in the 30Cs gradually decreases in the following order: PET > 30C > 30CB2 > 30CG2, and eventually vanishes with further incorporation of NDC. Figure 11b compares the XRD patterns before and after sample degradation. The broader half-width of the characteristic peaks of the degraded sample and the reduced peak intensity, according to the Scherrer equation, indicates a decrease in crystal size and cross-linking density. By comparing the effect of BTC and GC on degradation behavior, it was found that the degradation rate of 30CG2 was significantly higher than that of 30CB2, whereas there was little difference in the impact of the cross-linker on 30CNs.

Based on the degradation data and XRD results, there appear to be two factors that affect the degradation rate of PET/PEN/PCL copolyesters. First, since PCL is the main component undergoing degradation, its crystallinity can affect the degradation process. The highly crystalline nature could severely impede the ingress of reactive species into the solid bulk. [50] The decreased crystallinity of cross-linked PCL enhances the accessibility of the degradation medium to the degradation site, thereby accelerating the degradation rate. Second, the cross-linking of polymers reduces the mobility of molecular chains and the frequency of contact between the molecules and the degradation medium, resulting in a lower degradation rate. For the 30CNs, the amorphous morphology and higher cross-linking efficiency of BTC, as shown in the DMA section, lead to a slightly lower degradation rate of 30CNB2 compared to 30CNG2. As for 30Cs, the combined effects of higher cross-linking efficiency and lower crystallinity induced by GC lead to a higher degradation rate of 30GC2 than 30GB2. The degradation experiments demonstrate that the degradation efficiency of PET/PEN/PCL copolyesters can be moderately regulated by adjusting the degree of crystallinity and cross-linking.

## 4. Conclusions

A series of novel shape-memory polymers (SMPs) were synthesized through the one-pot polycondensation reaction, including PET/PCL (30Cs) and PET/PEN/PCLs (30CNs) with either planar (BTC) or non-planar (GC) cross-linker. The chemical structure of the materials was confirmed using infrared and NMR spectra, and NMR analysis revealed that 30CNs have a longer segment length and a more random distribution between hard and soft segments. The addition of PEN resulted in a significant increase in tensile strength and a transformation of the material from semi-crystalline to amorphous. DMA, tensile test, and intrinsic viscosity test results indicated that GC has a higher cross-linking efficiency than BTC for 30Cs, while the difference is not significant for 30CNs. However, for the amorphous SMP, 30CN, using a small amount of planar cross-linking agent for cross-linking can effectively improve the shape fixation rate (R_f_) and shape recovery rate (R_r_). The material with the highest tensile strength (57.1 MPa) and excellent shape-memory performance (R_r_(3) = 99.1%, R_f_(3) = 90.8%) was 30CNB2, which contained both PEN and planar cross-linking agent as rigid cross-linking nodes. This study provides a new strategy for the development of SMPs by adjusting the crystallization morphology and cross-linking forms. The prepared PET/PEN/PCLs have high strength, excellent shape-memory properties, good thermal stability, T_g_ nearing body temperature, and controllable biodegradability, making them suitable for use in smart textiles and biomedical materials.

## Figures and Tables

**Table 1:** The composition and molar ratio of PET/PEN/PCL copolyesters with cross-linkers.

Sample	Input						Output *			
	Hard Segment			Soft Segment ^#^	Cross-linker ^+^		Hard Segment			Soft Segment
	Acid or Ester		Alcohol				Acid or Ester		Alcohol	
	TPA	NDC	EG	CL	GC	BTC	TPA	NDC	EG	CL
PET	1	0	1.2	0	0	0	1	0	1	0
30C	1	0	1.2	0.51	0	0	1	0	0.65	0.30
30CG2	1	0	1.2	0.51	0.02	0	1	0	0.64	0.30
30CB2	1	0	1.2	0.51	0	0.02	1	0	0.66	0.36
30CN	0.67	0.33	1.2	0.55	0	0	0.67	0.32	0.68	0.34
30CNG2	0.67	0.33	1.2	0.55	0.02	0	0.67	0.32	0.67	0.36
30CNB2	0.67	0.33	1.2	0.55	0	0.02	0.67	0.32	0.67	0.36

* Output molar ratio is determined by the integral area of corresponding peak of each segment from NMR spectra. ^#^ The molar ratio of CL is calculated using the molecular weight of the repeating unit of PCL3000, which is M_cl_ = 114. ^+^ The output molar ratio could not be determined due to the content of cross-linker being too low to be detected from the NMR spectrum.

**Table 2:** Assignment of the signals through FT-IR of the synthesized polymers.

Wavenumber (cm^−1^)	Type of Vibration
3422	Overtone C=O
3063	Benzene CH vibration
2967, 2963	Amorphous methylene stretch, CH_2_
2899	Crystalline stretch, CH_2_
1720	Carbonyl stretch, C=O
1336, 1120, 871	Aromatic ring vibration
1239	Asymmetric stretch, C-O-C
1179, 764	Naphthalene ring vibration

**Table 3:** The chemical shifts of PET/PEN/PCL copolyesters and the structure parameter determined by ^1^H-NMR.

Sample	a	b, b_1_, b_2_, b_3_	c, c′_1_, c″_1_	c_2_, c′_2_, c_3_, c′_3_, d_1_, d′_1_	d	e	f, f′,g	h	X_EG′_	X_CL_	n	m + l	R
	8.8	8.1~8.3	4.9~5.1	4.4~4.8	4.25	2.5	1.8	1.5					
PET	-	1.00	1.00	-	-	-	-	-	0.00	-	-	-	-
30C	-	1.00	0.65	0.49	0.07	0.17	0.35	0.15	0.35	0.23	0.86	2.86	1.52
30CG2	-	1.00	0.64	0.50	0.08	0.17	0.35	0.15	0.36	0.23	0.83	2.78	1.56
30CB2	-	1.00	0.66	0.54	0.08	0.18	0.38	0.18	0.34	0.26	1.06	2.94	1.28
30CN	0.16	1.00	0.68	0.48	0.03	0.18	0.38	0.17	0.31	0.26	1.11	3.22	1.22
30CNG2	0.16	1.00	0.67	0.52	0.03	0.18	0.38	0.18	0.32	0.27	1.14	3.12	1.21
30CNB2	0.16	1.00	0.67	0.50	0.03	0.19	0.4	0.18	0.32	0.27	1.14	3.12	1.21
PCL	-	-	-	-	1.00	1.00	2.00	1.00	-	-	-	-	-

**Table 4:** The thermal properties of PET/PEN/PCL copolyesters.

Sample	DSC 1st Heating					DSC 1st Cooling		DSC 2nd Heating					DMA		TGA		X *	CD ^#^
	T_g_	T_cc_	ΔH_cc_	T_m_	ΔH_m_	T_c_	ΔH_c_	T_g_	T_cc_	ΔH_cc_	T_m_	ΔH_m_	T_g_	tanδ	T_d-5%_	Char		
	(°C)	(°C)	(J/g)	(°C)	(J/g)	(°C)	(J/g)	(°C)	(°C)	(J/g)	(°C)	(J/g)	(°C)	-	(°C)	(%)		
PET	72.9	126.5	3.8	251.4	38.5	173.4	−22.7	74.9	123.6	13.8	250.6	37.5	90.1	0.17	397.9	20.0	18.9	0
30C	34.2	124.2	3.4	183.1	22.8	111.9	−4.8	34.4	122.3	11.1	182.3	16.4	31.4	0.28	376.1	12.7	4.0	0
30CG2	38.9	125.5	1.2	172.1	14.1	120.2	−1.5	39.8	120.3	5.1	170.0	7.9	35.2	0.18	381.0	13.1	1.2	156
30CB2	36.2	124.4	2.6	177.0	17.5	116.9	−2.9	36.5	121.5	9.1	172.8	12.3	33.6	0.21	379.1	13.4	2.4	97
30CN	42.5	-	-	-	-	-	-	41.6	-	-	-	-	43.5	1.09	383.4	17.1	-	0
30CNG2	44.4	-	-	-	-	-	-	43.6	-	-	-	-	44.3	0.86	386.2	17.4	-	77
30CNB2	46.8	-	-	-	-	-	-	45.0	-	-	-	-	45.1	0.84	388.2	17.7	-	89
PCL	-	-	-	61.0	51.7	20.6	−44.8	-	-	-	60.7	51.1	38.4	0.09	330.5	2.6	32.1	0

* X: Crystallinity of PET and PET-based copolymers is calculated by dividing ΔH_c_ by 119.8 J/g, which is the enthalpy of 100% crystalline PET. Crystallinity of PCL is calculated by dividing ΔH_c_ by 139.5 J/g, which is the enthalpy of 100% crystalline PCL. ^#^ CD: Cross-linking density (mol/m^3^).

**Table 5:** Mechanical properties of the PET, PCL, and PET/PEN/PCLs and their intrinsic viscosities.

Sample	Tensile Strength	Elongation at Break	Yielding Strength	Young’s Modulus	[η]
	MPa	%	MPa	MPa	dL/g
PET	64.9	6.8	-	1064.8	0.59
30C	27.8	200.1	8.5	556.0	0.80
30CG2	35.8	139.8	-	151.2	0.94
30CB2	32.8	169.8	24.0	257.9	0.85
30CN	53.2	9.4	-	675.4	0.70
30CNG2	54.4	8.3	-	763.5	0.76
30CNB2	57.1	8.3	-	773.1	0.78
PCL	21.8	1201.0	22.8	89.3	1.07

**Table 6:** The shape recovery ratio (R_r_) and shape fixation ratio (R_f_) of PET/PEN/PCLs.

Sample	R_f_(1)	R_f_(2)	R_f_(3)	R_r_(1)	R_r_(2)	R_r_(3)
30C	93.0	93.9	93.9	85.2	93.0	94.9
30CG2	79.1	82.9	82.2	90.8	94.6	97.1
30CB2	85.1	88.2	89.3	90.9	94.4	96.7
30CN	84.2	87.4	89.3	87.0	93.9	96.1
30NG2	78.1	81.5	82.1	94.8	97.9	99.0
30NB2	84.3	88.2	90.8	96.0	97.9	99.1

## Data Availability

My data is accurate and reliable, and the experiment can be repeated. I promise that my information is fully available.
